# Establishing Local Diagnostic Reference Levels and Reference Curves for Thorax and Abdomen-Pelvis Paediatric CT Procedures

**DOI:** 10.15388/Amed.2025.32.1.12

**Published:** 2025-02-18

**Authors:** Rokas Dastikas, Antonio Jreije, Birutė Gricienė

**Affiliations:** 1 Faculty of Medicine, Vilnius University, Vilnius, Lithuania; 2 Vilnius University Hospital Santaros Klinikos, Vilnius, Lithuania; 3 Faculty of Medicine, Vilnius University, Vilnius, Lithuania Vilnius University Hospital Santaros Klinikos, Vilnius, Lithuania

**Keywords:** paediatric, diagnostic reference levels, diagnostic reference level curves, thorax computed tomography, abdomen-pelvis computed tomography, pediatrija, diagnostiniai atskaitos lygiai, diagnostinių atskaitos lygių kreivės, krūtinės KT, pilvo ir dubens KT

## Abstract

**Background:**

Computed tomography is a highly informative diagnostic tool, but its use poses the challenge of managing potentially high radiation exposure to patients. Children are particularly vulnerable to the harmful effects of ionizing radiation, and the growing use of paediatric *Computed Tomography* (CT) scans has been linked to an elevated lifetime risk of cancer and an increased mortality. The aim of this study was to evaluate local radiation exposure doses in paediatric thoracic and abdominal-pelvic CT exams, to establish *Diagnostic Reference Level* (DRL) curves, propose local diagnostic reference levels, and compare them with the existing literature and the European Guidelines on Diagnostic Reference Levels for Paediatric Imaging (PiDRL).

**Materials and Methods:**

A dataset of thoracic and abdominal-pelvic CT exams performed on children was analysed. Scan data entries were grouped according to the patient weight in the following intervals: 5 to 14 kg, 15 to 29 kg, 30 to 49 kg, and 50 to 79 kg. In each weight group, the minimum, first quartile, median, third quartile, and the maximum values of *Volumetric Computed Tomography Dose Index* (CTDI_vol_) and the *Dose Length Product* (DLP) were calculated. The relationship between CTDI_vol_, DLP, and the patient body weight was assessed by using exponential curves.

**Results:**

The local DRLs were established for thoracic CT exams, while, for abdominal-pelvic CT exams, the DRL curve was set as a substitute due to limited data. The proposed local DRL values for thoracic computed tomography examinations are 2.0, 2.4, 3.6, and 5.0 mGy for CTDI_vol_ and 40, 60, 116, and 156 mGy•cm for DLP in the corresponding weight groups of 5 to 14 kg, 15 to 29 kg, 30 to 49 kg, and 50 to 79 kg. The median values of CTDI_vol_ for paediatric abdominal-pelvic computed tomography were 2.8 mGy in the 5-to-14 kg weight group, 3.6 mGy in the 15-to-29 kg group, 4.8 mGy in the 30-to-49 kg group, and 7.9 in the 50-to-79 kg group. The median DLP values were 81, 127, 203, and 304 mGy•cm, respectively.

**Conclusions:**

The set local DRLs for thoracic and the median dose values in abdominal-pelvic CT exams are generally lower than the European DRLs. The derived DRL curves fulfil the same purpose as weight-group DRLs, serving as benchmarks for dose optimization.

## Abstract (it)

**Įvadas:**

Kompiuterinė tomografija (KT) yra labai informatyvi diagnostikos priemonė, tačiau jos naudojimas susijęs su pacientų patiriama potencialiai didele jonizuojančiosios spinduliuotės apšvita. Vaikai yra ypač jautrūs neigiamam jonizuojančiosios spinduliuotės poveikiui, todėl vis dažniau atliekami kompiuterinės tomografijos tyrimai vaikams didina vėžio riziką ir mirštamumą nuo jų. Šio tyrimo tikslas buvo įvertinti jonizuojančiosios spinduliuotės dozes vaikams, patiriamas atliekant krūtinės ląstos bei pilvo ir dubens organų kompiuterinės tomografijos tyrimus, nustatyti diagnostinių atskaitos lygių (DAL) kreives, pateikti vietinius diagnostinius atskaitos lygius krūtinės ląstos KT tyrimams ir palyginti juos su europiniais ir literatūroje pateiktais DAL.

**Medžiaga ir metodai:**

Analizuotas krūtinės bei pilvo ir dubens KT tyrimų, atliktų vaikams, duomenų rinkinys. Duomenų įrašai buvo grupuoti pagal paciento svorį šiais intervalais: nuo 5 iki 14 kg, nuo 15 iki 29 kg, nuo 30 iki 49 kg ir nuo 50 iki 79 kg. Kiekvienai svorio grupei buvo apskaičiuotos tūrinio kompiuterinės tomografijos dozės indekso (CTDI_vol_) ir dozės ilgio sandaugos (DLP) minimalios, pirmojo kvartilio, medianos, trečiojo kvartilio ir didžiausios vertės. CTDI_vol_, DLP ir paciento kūno svorio ryšys buvo įvertintas naudojant eksponentines kreives.

**Rezultatai:**

Vietiniai DAL buvo nustatyti krūtinės ląstos KT tyrimams, o pilvo ir dubens KT tyrimams DAL kreivė buvo nustatyta kaip vietinių DAL alternatyva esant ribotam KT tyrimų kiekiui. Siūlomos vietinės DAL vertės krūtinės ląstos KT tyrimams yra 2,0, 2,4, 3,6 ir 5,0 mGy pagal CTDI_vol_ ir 40, 60, 116 ir 156 mGy•cm pagal DLP atitinkamose svorio grupėse nuo 5 iki 14 kg, nuo 15 iki 29 kg, nuo 30 iki 49 kg ir nuo 50 iki 79 kg. Vaikų pilvo ir dubens organų KT vidutinės CTDI_vol_ reikšmės buvo 2,8 mGy 5–14 kg svorio grupėje, 3,6 mGy 15–29 kg grupėje, 4,8 mGy 30–49 kg grupėje ir 7,9 50–79 kg grupėje. Vidutinės DLP reikšmės atitinkamai siekė 81, 127, 203 ir 304 mGy•cm.

**Išvados:**

Nustatyti vietiniai krūtinės ląstos DAL ir dozės vertės medianos atliekant pilvo ir dubens KT tyrimus paprastai yra mažesnės nei europiniai DAL. DAL kreivės atlieka tą pačią funkciją kaip ir svorio grupių DAL, kuriais remiantis dažniausiai vertinama patiriama apšvita.

## Background

*Computed Tomography* (CT) is a highly informative diagnostic tool in modern medicine, yet its use comes with the critical challenge of managing potentially high radiation exposure to patients. Over the past two decades, the use of CT has almost doubled, resulting in over 400 million annual examinations performed globally [1]. Despite comprising only 10 percent of all diagnostic radiological procedures, CT is responsible for over 60 percent of all collective effective dose caused by all imaging modalities [1,2].

This trend has also been observed in Lithuania: in 2023, approximately 520,000 CT examinations were performed, which is an almost quadruple increase since 2006. Additionally, while paediatric CT imaging constitutes only a small fraction of all radiological investigations, the number of investigations has increased by 50 percent over the course of three years with head, chest, abdomen-pelvis CT examinations being the most commonly performed varieties [3,4].

Excessive radiation exposure may lead to two types of tissue damage: deterministic effects, otherwise known as tissue reaction, which are characterised by acute cell or tissue damage caused by reaching a particular dose threshold and with the severity proportional to the acquired dose, and stochastic effects, causing malignant disease or hereditary changes of undeterminable severity (Clement, 2017). Children are more susceptible to the stochastic effects compared to adults due to their anatomical differences, higher tissue sensitivity, particularly of the red bone marrow, breast, thyroid, and lungs, as well as longer life expectancy, making presentation of a malignant disease more likely [5]. An increasing number of studies and reports show that the use of paediatric CT is associated with an increase in the lifetime cancer risk and mortality, particularly if the examinations are performed at a very young age [6–9].

The risk associated with radiation exposure increases with the number of repeated examinations and is directly proportional to the cumulative radiation dose [10,11]. This underscores the importance of assessing paediatric patient exposure during diagnostic and interventional radiological procedures so that to optimize doses and minimize the potential adverse health effects. *Diagnostic Reference Levels* (DRLs) are essential tools for dose monitoring, generally set at the 75^th^ percentile of the median dose distribution for a specific examination or procedure. Exceeding these DRLs prompts further investigation and optimization of radiation practices [12]. DRLs have been a part of the European legislation since 1997, and reiterated in 2013 with the requirement that all member states should establish and regularly review and update their national DRLs [12,13].

However, establishing national diagnostic reference levels for paediatric patients is challenging and inconsistent due to the relatively small number of performed procedures, as well as large variations in the patients’ age, weight, and size. Consequently, there is a limited availability of publications, data, and guidance from authoritative radiation protection bodies [13]. While the European Commission has introduced the *European Guidelines on Diagnostic Reference Levels for Paediatric Imaging* (PiDRL), Lithuania has only established national DRLs for head CT imaging, with no national reference levels currently defined for chest or abdominopelvic CT scans [14].

When national DRLs are not established or when different protocols, methods and new technological advancements are used in imaging practices, local DRLs, which can be set for use in a single large or several smaller healthcare institutions, are particularly useful [12]. Local DRLs may also be established when the use of national DRLs does not factor in the specific needs of highly specialized institutions, for example, in oncological centers [13].

In case of limited patient data, DRL curves, a mathematical fit to radiation dose data, can offer a valuable alternative for defining the relationship between the patient weight and the radiation dose. When establishing DRL curves, an equivalent diameter or weight often substitutes for thickness, and the radiation dose is evaluated by using curve fitting techniques. DRL curves express dose quantities as a continuous function of a grouping parameter, provided the data show a clear relationship between the two [13]. This approach addresses the challenge of poor statistics by eliminating the need to gather adequate dose data for discrete patient groups. [15].

The aim of this study was to evaluate local radiation exposure doses in paediatric thoracic and abdominal-pelvic computer tomography examinations at a tertiary-level hospital, establish DRL curves, propose local diagnostic reference levels and compare them with existing literature and European Guidelines on DRLs for Paediatric Imaging (PiDRL).

## Materials and Methods

### Data collection

A dataset of thoracic and abdominal-pelvic CT examinations performed on children aged 0 to 17 was retrospectively analysed. All scans were acquired at Vilnius University Hospital Santaros Clinics between 2020 and 2022 by using a *Siemens Somatom Sensation 64 CT* scanner. The patient data, including their weight, age, and the scanned area as well as the information on the number of scan series, scan parameters, and the resulting dose in the *Volumetric Computed Tomography Dose Index* (CTDI_vol_) and the *Dose Length Product* (DLP) were collected. A 32 cm phantom was used to determine, calibrate and check the dose quantities. Multi-phase examinations were not excluded from this analysis, and the average values of CTDI_vol_ and DLP for plain and contrast enhanced scans were used.

### Setting local DRLs

Scan data entries were grouped according to the patient weight in the following intervals: 5 to 14 kg, 15 to 29 kg, 30 to 49 kg, and 50 to 79 kg. These weight bands are suggested by PiDRL [13] and endorsed by the International Commission on Radiological Protection [12]. In each weight group, the minimum, the first quartile, the median, the third quartile, and the maximum values of CTDI_vol_ and DLP were calculated for both thoracic and abdominal-pelvic CT examinations. The local DRLs were defined as the third quartile values of the distributions. For the descriptive analysis, entries with the patient weight falling outside of the specified ranges were excluded.

All data entries were used to assess the relationship between CTDI_vol_, DLP, and the patient body weight, by using the Spearman rank-order correlation coefficient and exponential curves. The decision to employ exponential curves over linear relationship models is based on the basic physical properties of X-rays, where the photon beams are attenuated exponentially over the thickness of the patients’ bodies [16]. The coefficient for the exponential curves, expressed as *y=ae^kx*, where *x* is the body weight of the patient, and *y* is the radiation quantity of either CTDI_vol_, or DLP, were derived by fitting an exponential trendline onto the datapoints to obtain the function growth rate coefficient *k*. The initial value *a* was calculated for each scan, and the median and the third quartile of the *a* values were identified. The median *a* value was used to express the median DLP and CTDI_vol_ curves, and the third quartile *a* value was used to define the DRL curves.

Statistical analysis was performed by using *R* and *Microsoft Excel* software.

A literature analysis was performed in the *PubMed* database by using the Medical Subject Heading terms for Infant, Child, Adolescent, X-Ray Computed Tomography, and Diagnostic Reference Levels. Publications published between 2014 and 2024, using DLP and CTDI_vol_ for the patient dose evaluation, proposing local, national, or regional DRLs for thoracic or abdominal-pelvic examinations, and using patient weight as the primary method of grouping patient examinations, were included for this review.

## Results

A total of 114 CT examinations were included in this study. Thoracic CT scans accounted for the majority of these examinations, with 85 procedures performed, while 29 patients underwent abdominal-pelvic CT scans. Among the thoracic CT scans, 29 examinations (34%) involved multiple scan series, whereas 26 abdominal-pelvic scans (90%) were conducted as multi-series investigations. A constant tube voltage of 120 kVp was maintained for both types of examinations. The median tube current value of 94 mA (interquartile range 56–140 mA) was used in thoracic CT and 107 mA (interquartile range 40–199 mA) for abdominopelvic CT. A filtered back projection reconstruction algorithm was used in all scans

### Thoracic CT examinations

The median values of CTDI_vol_ for paediatric thoracic CT were 1.6 mGy in the 5-to-14 kg weight group, 2.0 mGy in the 15-to-29 kg group, 3.4 mGy in the 30-to-49 kg group, and 4.5 in the 50-to-79 kg group. The median DLP values were 35, 51, 102, and 143 mGy•cm, respectively. Additional data are provided in Table 1 and Table 2.

**Table 1:** CTDIvol values for paediatric thoracic CT examinations by weight group

Weight group	Number of patients	CTDI_vol_, mGy				
		Minimum	1^st^ quartile	Median	3^rd^ quartile (local DRL)	Maximum
5 to 14 kg	6	1.4	1.5	1.6	2.0	2.2
15 to 29 kg	23	1	1.7	2.0	2.4	3.8
30 to 49 kg	28	1.3	3.0	3.4	3.6	4.4
50 to 79 kg	22	3.6	4.1	4.5	5.0	6.3

**Table 2:** DLP values for paediatric thoracic CT examinations by weight group

Weight group	Number of patients	DLP, mGy•cm				
		Minimum	1^st^ quartile	Median	3^rd^ quartile (local DRL)	Maximum
5 to 14 kg	6	33	34	35	40	49
15 to 29 kg	23	23	40	51	60	93
30 to 49 kg	28	25	88	102	116	163
50 to 79 kg	22	94	133	143	156	227

The proposed local DRL values for thoracic CT examinations are 2.4, 3.6, and 5.0 mGy for CTDI_vol_ and 60, 116, and 156 mGy•cm for DLP in the corresponding weight groups of 15 to 29 kg, 30 to 49 kg, and 50 to 79 kg.

A strong positive correlation was observed between the patient weight and both CTDI_vol_ (ρ=0.86, *p*<0.001) and DLP (ρ=0.90, *p*<0.001).

**Figure 1:**
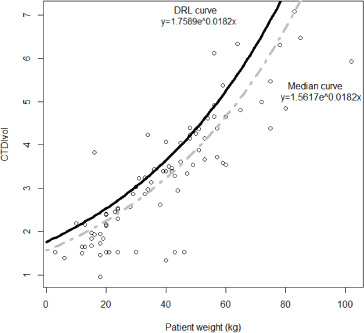
Median value (grey dot line) and DRL (solid black line) reference curves based on CTDI_vol_ for thoracic CT examinations.

**Figure 2:**
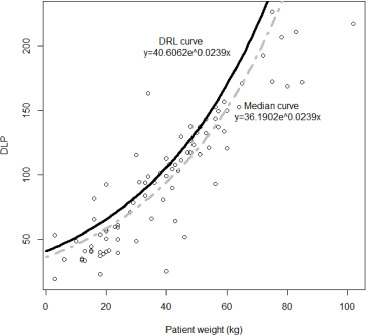
Median value (grey dot line) and DRL (solid black line) reference curves based on DLP for thoracic CT examinations.

An exponential reference curve was fitted onto a scatterplot between the patient weight and both CTDI_vol_ (*R^2^=0.70*) and DLP (*R^2^=0.72*). The derived DRL and median value curves are expressed and visualized in Figures 1 and 2, where *x* is the weight of the patient, and *y* is the radiation quantity of either CTDI_vol_ or DLP.

### Abdominal-pelvic CT examinations

The median values of CTDI_vol_ for paediatric abdominal-pelvic CT were 2.8 mGy in the 5-to-14 kg weight group, 3.6 mGy in the 15-to-29 kg group, 4.8 mGy in the 30-to-49 kg group, and 7.9 in the 50-to-79 kg group. The median DLP values were 81, 127, 203, and 304 mGy•cm, respectively. The third quartile values of CTDI_vol_ for paediatric abdominal-pelvic CT were 3 mGy in the 5-to-14 kg weight group, 4.1 mGy in the 15-to-29 kg group, 5.9 mGy in the 30-to-49 kg group, and 8.3 in the 50-to-79 kg group. The third quartile DLP values were 87, 160, 203, and 428 mGy•cm, respectively.

**Figure 3:**
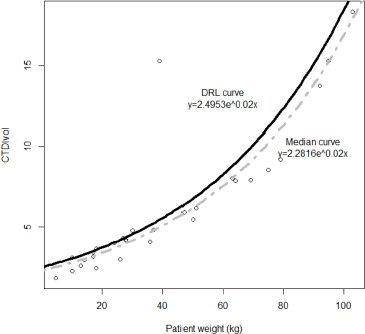
Median value (grey dot line) and DRL (solid black line) reference curves based on CTDI_vol_ for abdominal-pelvic CT examinations.

**Figure 4:**
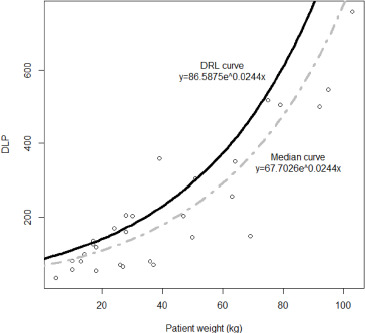
Median value (grey dot line) and DRL (solid black line) reference curves based on DLP for abdominal-pelvic CT examinations.

Local DRLs could not be established for abdominal-pelvic procedures based on the weight group due to an insufficient number of patients per weight group, with the count falling below the 20-patient threshold recommended by the European Guidelines. Instead, DRL reference curves were set (Figures 3 and 4).

A strong positive correlation is observed between both patient weight and CTDI_vol_ (ρ=0.95, *p*<0.001) and patient weight and DLP (ρ=0.81, *p*<0.001). An exponential reference curve was fitted onto a scatterplot between the patient weight and both CTDI_vol_ (*R^2^=0.78*) and DLP (*R^2^=0.83*). The derived DRL and median value curves are expressed and visualized in Figures 3 and 4, where x is the weight of the patient, and y is the radiation quantity of either CTDI_vol_ or DLP.

## Discussion

Paediatric CT scans are performed far less frequently than those on adult patients, thereby making it challenging to collect a sufficient number of examinations. This difficulty is further exacerbated when the already limited data is further divided into subgroups. Additional factors that hinder data collection include the underutilization of automated dose monitoring and management systems, as well as the lack of well-developed dose audit surveys and systems with carefully predefined parameters [13,17,18]. Furthermore, up until the publication of guidelines by both the International Commission on Radiological Protection [12] and the European Commission [13], the process of establishing DRLs lacked uniformity, primarily on the use of dosimetric phantoms and the grouping of patients [19,20].

The majority of previously published DRLs for paediatric thoracic and abdominal-pelvic CT examinations grouped the examination by the patients’ age [13,19,20]. However, this parameter is suboptimal when assessing the radiation exposure because it does not take into account the rapid growth of infants, and potentially ignores the size difference between children of the same age, which makes physically larger patients even more susceptible to increased doses of radiation to obtain images of required quality [13,20,21]. Even if the age ranges are converted into weight ranges, up to a quarter of the patients might be inappropriately categorized [15]. Additionally, the patients’ weight shows a stronger correlation with the size of the patient rather than their age, making it the preferred patient characteristic for evaluating radiation exposure [22–24]. These reasons prompted the use of weight for patient categorization in this study. While SSDE and water-equivalent diameter are currently more accurate and direct representations of the patient size compared to CTDI_vol_ or DLP, not all scanning equipment and software are able to provide these parameters automatically, which makes its current utilisation more limited [12,13,21].

Inclusion and assessment of multi-phase examinations in the establishment of DRLs varied significantly in recent publications: some included multi-phase examinations and assessed the total DLP value along with the highest CTDI_vol_, value, as recommended by the IRCP guidelines, which mainly focus on dose monitoring of adult patients [12,15], while some assessed only single-phase examinations [21,22,25], and others did not specify this information [18,26]. While the European guidelines suggest setting up DRL values based solely on values from scan series of single-phase examinations [13], a significant portion of all chest and abdomen-pelvis CT investigations in this study are multi-series examinations. Therefore, all scan series were included, and the average values of DLP and CTDI_vol_ were assessed so that to estimate the expected DRL quantities for a single phase of an examination

When comparing our proposed local DRLs for thoracic CT examinations with the European DRLs, it can be seen that the values generally align with those outlined in the guidelines [13]. Both the obtained CTDI_vol_ values and the DLP values tend to be similar or lower, especially in the 50-to-79 kg group, where DRLs based on DLP are up to 30 percent lower. None of the calculated median values for either thoracic or abdominal-pelvic examinations exceeded the European DRLs.

**Figure 5:**
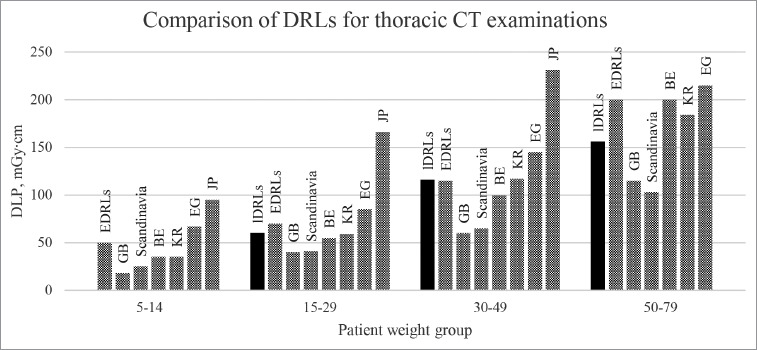
Comparison of DRLs by DLP for thoracic CT examinations. lDRLs – local DRLs in this study, EDRLs – European DRLs, EG – Egyptian national DRLs, KR – South Korean local DRLs, JP – Japanese national DRLs, GB – national DRLs in the United Kingdom, Scandinavia – regional Scandinavian DRLs.

There is a limited number of publications on dose assessment and local DRL establishment in paediatric CT patients. While reviewing the current literature, according to the searching criteria for patient grouping and the body region of CT procedures, only five studies were identified which allowed for direct comparison with our set thoracic DRLs. A visual representation of the DRL values from different countries is provided in Figure 5. The Egyptian national DRLs and the local DRLs across several institutions in South Korea were the closest to the ones proposed in this study, with the average difference between DLP values not exceeding 22 percent [22,25]. The Japanese survey proposed the highest DRLs among all the studies, with DLP values nearly double those in this study [26]. The national DRL survey in the United Kingdom and a regional DRL study in Scandinavia achieved significantly lower DRLs, averaging around 40% less than the values obtained in this work [17,18].

One potential reason for the significant differences observed between the studies may lie in the examination protocols used. Whereas, in this study, a tube voltage of 120 kVp was used for most scans, the study by Worrall et al. in the United Kingdom exhibited a greater variability in thoracic CT protocols, with tube voltage values ranging from 70 to 120 kVp, and with 80kVp being the most frequently used option [18]. Lowering of the tube voltage can reduce the radiation dose to patients while enhancing the contrast of soft tissue structures and contrast agents which are both highly desirable outcomes in paediatric radiology [27,28]. The increased noise caused by the reduction of the tube voltage can be reduced by appropriately adjusting the tube output value [29]. Therefore, in order to reduce the radiation exposure of children, the possibility of lowering the tube voltage in our facility should be considered in the future.

While the establishment of DRLs has proven to be an effective means of dose optimization in paediatric CT [30], it is important to note that a reduction of exposure doses below the DRLs does not necessarily indicate a fully optimized procedure [12]. Moreover, as DRLs are not intended to be used on individual patients, a troubling tendency of using the DRL value as the dose limits can hinder proper optimization. This issue is particularly pronounced in physically larger patients, where adherence to such limits may compromise the imaging quality as well as the diagnostic accuracy [31]. Therefore, ICRP recommends that, when establishing national DALs, the median values of the radiation quantity doses should also be indicated in order to serve as an additional reference point for optimization. If institutional doses are below this value, the optimization efforts should be focused on improving the quality of images, since the diagnostic clarity in medical imaging is paramount [12].

The establishment of DRLs for specific indications should also be considered in patient dose optimization as doses can differ significantly between different indications [13]. Indication and disease specific protocols have proven to be effective in substantial patient dose reduction while maintaining the diagnostic accuracy [32,33]. Other disease specific protocols may provide more informative imaging by switching to another modality without an increase in the dose exposure [34].

The implementation of DRL curves may offer significant advantages in the clinical practice. Compared to the prevalent method of using age or weight groups, DRL curves require substantially fewer scans to establish the reference levels (i.e., at least 10 patients per curve), while providing hospitals and specialists with an efficient tool to assess their use of CT in paediatric examinations and the associated radiation exposure. Additionally, DRL curves enable effective monitoring of the dose quantities for specific protocols or indications, particularly when the establishment of traditional DRLs is unfeasible due to a low number of examinations. By providing a clear visual representation, these curves allow clinicians to quickly and easily determine whether the radiation dose from an investigation falls within the acceptable thresholds. Moreover, the continuous scale provided by DRL curves supports a more individualized approach to imaging, facilitating the selection of optimized imaging parameters tailored to each patient’s needs, thereby enhancing dose optimization and ensuring safer radiological practices [15,23,35].

The use of DRL curves in the clinical practice is relatively straightforward. Whenever regular dose audits are performed, if data from at least 10 patients – regardless of their weight – are available, a new third quartile dose quantity curve can be fitted. This new curve should then be visually plotted against the established DRL curve to determine if the doses do not exceed the DRLs. Any outliers can be easily determined by comparing their individual dose quantities with the DRL value obtained by inserting their weight into the formula. If these audits show that the DRLs are repeatedly exceeded, additional means of dose optimization should be considered in the imaging practices.

## Conclusion

Local DALs were determined for each weight group based on the values of the 3^rd^ quartile of the patient dose quantity distribution. For paediatric chest CT examinations, DRLs based on CTDIvol are 2.4 mGy in the 15–29 kg weight group, 3.6 mGy in the 30–49 kg group, and 5.0 mGy in the 50–79 kg group. DRLs according to DLPs are set as 40, 60, 116 and 156 mGy∙cm, respectively. Accompanying DRL exponential curves were also set. Weight band DRLs for abdominal-pelvic CT studies were not able to be determined. Instead, DRL curves were calculated and visually expressed, and they serve the same function to assess and estimate paediatric exposure doses for children of different weights.

Weight-based DRL curves represent a practical and effective approach, particularly as a supplement to the traditional DRLs in scenarios where data are limited. In this study, local DRLs were successfully established for thoracic procedures, while DRL curves were utilized as a substitute for abdominal-pelvic procedures due to insufficient data.

The derived DRL curves could fulfil the same purpose as weight-group DRLs, serving as benchmarks for dose optimization. A dose index for an individual patient above the curve is not inherently concerning; however, if the majority of patient dose indices consistently exceed the DRL curve, further investigation is warranted, and dose adjustments should be considered wherever feasible.

The primary advantage of DRL curves lies in their clinical applicability. In situations with low examination frequencies, the time required to gather sufficient data to establish DRL values for multiple weight groups can be prohibitively long. DRL curves, by contrast, enable faster dose comparisons with fewer data points, thereby making them a valuable tool for optimizing radiation doses in the clinical practice.
